# Memantine use and risk of cardiac arrhythmias in Alzheimer dementia: a report from a global federated research network

**DOI:** 10.1093/europace/euaf299

**Published:** 2025-11-20

**Authors:** Laurent Fauchier, Thibault Lenormand, Lisa Lochon, Arnaud Bisson, Sandrine Venier, Pascal Defaye

**Affiliations:** Department of Cardiology, Centre Hospitalier Universitaire Trousseau et Faculté de Médecine, INSERM UMR1327 ISCHEMIA, Université François Rabelais, Avenue de la République, Tours 37044, France; Department of Cardiology, Centre Hospitalier Universitaire Trousseau et Faculté de Médecine, INSERM UMR1327 ISCHEMIA, Université François Rabelais, Avenue de la République, Tours 37044, France; Department of Cardiology, Centre Hospitalier Universitaire Trousseau et Faculté de Médecine, INSERM UMR1327 ISCHEMIA, Université François Rabelais, Avenue de la République, Tours 37044, France; Department of Cardiology, Centre Hospitalier Universitaire Trousseau et Faculté de Médecine, INSERM UMR1327 ISCHEMIA, Université François Rabelais, Avenue de la République, Tours 37044, France; Department of Cardiology, Centre Hospitalier Universitaire de Grenoble Alpes, et Université Grenoble Alpes, INSERM U1039, LRB, Grenoble, France; Department of Cardiology, Centre Hospitalier Universitaire de Grenoble Alpes, et Université Grenoble Alpes, INSERM U1039, LRB, Grenoble, France

**Keywords:** Alzheimer’s disease, Memantine, Atrial fibrillation, Ventricular arrhythmias, Conduction disturbances

## Introduction

Dementia is a major global health concern, and Alzheimer’s disease (AD) is frequently complicated by cardiovascular comorbidities, including atrial fibrillation (AF) and other arrhythmias.^1^ Memantine, an N-methyl-D-aspartate (NMDA) receptor antagonist approved for moderate-to-severe AD, may influence cardiac excitability through mechanisms distinct from conventional antiarrhythmic drugs. Preclinical studies suggest antiarrhythmic effects, and limited clinical data have reported lower AF incidence among memantine users, though evidence remains limited.^2–4^ Using the TriNetX global federated research network, we evaluated the association between memantine use and the risk of cardiac arrhythmias in a large, real-world cohort of patients with AD.

## Methods

Data were obtained from the TriNetX Global Collaborative Network, a federated database of anonymized electronic medical records from more than 150 million patients across 156 healthcare organizations. Adults aged 50–90 years with a diagnosis of AD (ICD-10 code G30) between 2010 and 2024 were included. Patients with at least two memantine prescriptions over >6 months formed the memantine group; those with ≥2 AD diagnoses for >6 months but no memantine use served as controls. The index date was defined as the second qualifying event (prescription or diagnosis). Follow-up began one day post-index and continued up to three years to limit protopathic bias. Primary outcomes were incident AF, ventricular tachycardia/fibrillation (VT/VF), cardiac arrest, and conduction disturbances (AV or bundle branch block). Secondary outcomes included syncope, acute myocardial infarction (MI), heart failure (HF) hospitalization, and death. Propensity score matching (1:1, calliper 0.1 SD) was applied across >60 demographic and clinical variables. Covariate balance was assessed using standardized mean differences (SMD). Hazard ratios (HRs) and 95% confidence intervals (CIs) were estimated by Cox models; risk ratios (RRs) were reported for outcomes violating proportional hazard assumptions (Schoenfeld residual test). Subgroup analyses compared outcomes by coronary artery disease (CAD) or HF status. The analysis was conducted in compliance with institutional and regulatory guidelines for retrospective, anonymized research.

## Results

After inclusion and matching, 183,494 patients with AD were identified, of whom 61 912 received memantine. Propensity score matching yielded two well-balanced cohorts of 60 689 patients each (SMD <0.1 for all covariates)(*Table 1*). During a follow-up of 1.8 ± 1.1 years (median 1.9), memantine use was associated with modest but statistically significant lower risks of AF (HR 0.94, 95%CI 0.89–0.99; *P* = 0.02) and VT/VF (HR 0.85, 95%CI 0.74–0.99; *P* = 0.03). A borderline association was observed for atrioventricular block (HR 0.92; *P* = 0.05), whereas bundle branch block and cardiac arrest did not differ. Syncope occurred slightly more frequently among memantine users (HR 1.08; *P* = 0.01) (*Table 1*).

**Table 1:** Baseline characteristics and outcomes of patients after propensity score matching

	AD, Memantine	AD, no Memantine	Std diff. (%)
	(*n* = 60 689)	(*n* = 60 689)	
*Demographics*			
Age (years), mean ± SD	78.3 +/- 8.2	78.4 +/− 8.8	1.6
Men, *n* (%)	20 985 (34.6%)	20 680 (34.1%)	1.1
White, *n* (%)	42 330 (69.7%)	42 480 (70%)	0.5
Black or African American, *n* (%)	5828 (9.6%)	6146 (10.1%)	1.8
Asian, *n* (%)	2899 (4.8%)	3038 (5%)	1.1
Hispanic or Latino, *n* (%)	3233 (5.3%)	3242 (5.3%)	0.1
Socioeconomic and psychosocial circumstances, *n* (%)	2383 (3.9%)	2459 (4.1%)	0.6
*Risk factors*			
Hypertension, *n* (%)	35 728 (58.9%)	36 371 (59.9%)	2.2
Diabetes mellitus, *n* (%)	12 761 (21%)	13 189 (21.7%)	1.7
Smoker, *n* (%)	8084 (13.3%)	8227 (13.6%)	0.7
Overweight or obesity, *n* (%)	4938 (8.1%)	5181 (8.5%)	1.4
Dyslipidaemia, *n* (%)	32 587 (53.7%)	33 622 (55.4%)	3.4
Alcohol related diagnoses, *n* (%)	947 (1.6%)	941 (1.6%)	0.1
*Cardiovascular comorbidities*			
Heart failure, *n* (%)	3874 (6.4%)	4022 (6.6%)	1
Coronary artery disease, *n* (%)	9361 (15.4%)	9544 (15.7%)	0.8
Myocardial infarction, *n* (%)	1580 (2.6%)	1648 (2.7%)	0.7
Ischaemic stroke, *n* (%)	4547 (7.5%)	4694 (7.7%)	0.9
*Non-cardiovascular comorbidities*			
Kidney disease, *n* (%)	11 303 (18.6%)	11 615 (19.1%)	1.3
COPD, *n* (%)	3977 (6.6%)	4177 (6.9%)	1.3
Sleep apnoea syndrome, *n* (%)	5588 (9.2%)	5642 (9.3%)	0.3
Peripheral vascular disease, *n* (%)	2881 (4.7%)	3365 (5.5%)	3.6
Previous cancer, *n* (%)	16 255 (26.8%)	16 588 (27.3%)	1.2
*Laboratory tests and examinations*			
Systolic BP (mm Hg), mean ± SD	130.5 +/− 20.3	130.6 +/− 20.6	0.5
Diastolic BP (mm Hg), mean ± SD	71.1 +/− 11.5	71.2 +/− 11.8	1.2
Heart rate, mean ± SD	72.0 +/− 13.3	72.8 +/− 13.3	5.7
Body mass index (kg/m2), mean ± SD	25.7 +/− 5.1	25.5 +/− 5.2	4.1
Total cholesterol (mg/dL), mean ± SD	178.2 +/− 45.4	178.4 +/− 46.2	0.5
Estimated GFR (MDRD, mL/min), mean ± SD	66.6 +/− 22.7	67.4 +/− 24.0	3.4
Haemoglobin A1c (%), mean ± SD	6.2 +/− 1.3	6.2 +/− 1.4	3.8
BNP, ng/L, mean ± SD	252.8 +/− 1102.7	299.0 +/− 1344.3	3.8
NT-proBNP, ng/L, mean ± SD	1599.0 +/− 4473.1	1515.9 +/− 4509.3	1.9
LVEF, mean ± SD	60.9 +/− 10.6	60.5 +/− 11.2	4
*Medications*			
Beta Blockers, *n* (%)	18 615 (30.7%)	19 096 (31.5%)	1.7
Calcium Channel Blockers, *n* (%)	15 670 (25.8%)	16 265 (26.8%)	2.2
ACE Inhibitors, *n* (%)	12 934 (21.3%)	13 227 (21.8%)	1.2
Angiotensin II Inhibitors, *n* (%)	10 538 (17.4%)	10 715 (17.7%)	0.8
ARNI, *n* (%)	100 (0.2%)	97 (0.2%)	0.1
MRA, *n* (%)	2272 (3.7%)	2280 (3.8%)	0.1
Diuretics, *n* (%)	15 556 (25.6%)	16 055 (26.5%)	1.9
Lipid lowering drugs, *n* (%)	29 627 (48.8%)	29 660 (48.9%)	0.1
Glucose-lowering therapy, *n* (%)	11 172 (18.4%)	11 604 (19.1%)	1.8
Antiplatelet therapy, *n* (%)	20 881 (34.4%)	20 947 (34.5%)	0.2

Clinical outcomes (FU 1.8 ± 1.1 years, median 1.9, IQR 2.3)				
	Number of events (yearly rate, %)		Hazard ratio (95% CI)	P value
Death	10 808 (8.63)	12 286 (10.28)	0.880 (0.859–0.900)^a^	<0.0001
Death, possibly sudden	2445 (2.22)	2797 (2.76)	0.874 (0.829–0.921)^a^	<0.0001
Incident AF	2635 (2.23)	2553 (2.38)	0.935 (0.886–0.988)	0.02
VT/VF	342 (0.29)	363 (0.34)	0.852 (0.735–0.988)	0.03
Cardiac arrest	409 (0.35)	380 (0.36)	0.972 (0.845–1.117)	0.69
VT/VF/Cardiac arrest	699 (0.60)	693 (0.65)	0.910 (0.819–1.011)	0.08
AV block	1117 (0.93)	1101 (1.04)	0.921 (0.847–1.001)	0.05
Incident BBB	1167 (0.99)	1078 (1.00)	0.978 (0.900–1.062)	0.6
Syncope	3048 (2.92)	2544 (2.70)	1.077 (1.022–1.135)	0.01
Acute MI	238 (0.20)	244 (0.23)	0.884 (0.740–1.057)	0.18
Hospitalization for HF	1129 (0.82)	1225 (0.95)	0.861 (0.794–0.934)	<0.0001

Values are *n* (%) or mean ± SD. ACE, angiotensin converting enzyme; AD, alzheimer dementia; AF, atrial fibrillation; ARNI, angiotensin receptor-neprilysin inhibitor; BBB, bundle branch block; BNP, Brain Natriuretic Peptide; BP, blood pressure; COPD, Chronic obstructive pulmonary disease; GFR, glomerular filtration rate; HF, heart failure; ICD, implantable cardioverter defibrillator; LVEF, left ventricular ejection fraction; MI, myocardial infarction; MRA, mineralocorticoid receptor antagonist; NT- proBNP, *n*-Terminal Prohormone Brain Natriuretic Peptide; SD, standard deviation; VT, ventricular tachycardia; VF, ventricular fibrillation.

^a^Risk ratio is presented instead of hazard ratio since the proportional hazard assumption was violated.

No significant difference was observed for acute MI, but hospitalizations for HF were lower with memantine (HR 0.86, 95%CI 0.79–0.93; *P* < 0.001). All-cause mortality (RR 0.88, 95%CI 0.86–0.90) and possibly sudden death (RR 0.87, 95%CI 0.83–0.92) were less frequent.

Subgroup analyses showed consistent associations across patients with and without CAD or HF. All *P*-values for interaction were nonsignificant except for mortality, where a significant interaction was found (*P* = 0.02). Overall, memantine’s associations with arrhythmic and cardiovascular outcomes were broadly similar regardless of underlying cardiac status.

## Discussion

In this large real-world cohort of patients with AD, memantine use was associated with modestly lower risks of atrial and ventricular arrhythmias, HF hospitalization, and mortality, without excess of conduction disturbances or MI. These associations were consistent across subgroups with and without CAD or systolic HF. The modest effects, although statistically significant, raise questions about their clinical relevance at the individual level, but may still be important in the context of high AF incidence in AD populations. Syncope occurred slightly more frequently with memantine, warranting further evaluation.

Preclinical studies suggest that N-methyl-D-aspartate (NMDA) receptor antagonists such as memantine may modulate cardiac excitability.^5^ NMDA receptors are expressed in myocardial tissue and autonomic ganglia, and their overactivation has been linked to arrhythmogenesis through altered calcium handling, oxidative stress, and autonomic imbalance.^6^ Our findings extend these insights to clinical practice, showing that NMDA receptor blockade may translate into lower arrhythmic event rates without proarrhythmic or extracardiac toxicity.

A few observational studies and registry analyses suggested a lower incidence of AF among memantine users, but duration of follow-up was unclear and confounding was possible.^4^ Our analysis distinguishes between syncope and AV block, cardiac arrest or VT/VF, emphasizing possible differences in mechanisms and ascertainment. Syncope or reflex-like episodes are common in AD and may lead to misclassification, whereas VT/VF represents confirmed arrhythmias.^7^ This may confirm prior reports suggesting that memantine may predispose to reflex syncope rather than conduction disturbances.^8^ Memantine’s associations with lower risks of HF hospitalization and MI may reflect broader cardiovascular effects, possibly through modulation of neurohormonal or microvascular pathways.^3^ Notably, unlike conventional antiarrhythmics, memantine showed no safety concerns in patients with structural heart disease.

This suggests that memantine may exert modest but favourable cardiovascular effects in AD, warranting prospective confirmation in randomized trials. Traditional antiarrhythmic drug options are limited in AD because of age, comorbidity burden, and concerns regarding tolerability or drug–drug interactions.^9^ Identifying safe agents with antiarrhythmic potential in this vulnerable population is therefore of high clinical importance.

This observational study has several limitations and deserves cautious interpretation. Residual confounding cannot be excluded despite extensive matching, and unmeasured factors such as frailty or cognitive severity may have influenced both memantine use and clinical events. Outcomes were identified from diagnosis codes rather than adjudicated clinical or electrocardiographic data, which may lead to misclassification or underestimation of true event rates. Information on dose, adherence, and arrhythmia burden was unavailable, and causality cannot be inferred.

Prospective randomized trials are needed to definitively test whether memantine reduces arrhythmic and cardiovascular events. The multicentre, randomized, double-blind, placebo-controlled STOP-AF study is currently exploring the efficacy and safety of memantine to treat patients with frequent and symptomatic atrial premature contractions.^10^

## Conclusion

In this large, propensity-matched cohort of patients with Alzheimer’s disease, memantine use was associated with modest but significantly lower risks of atrial and ventricular arrhythmias and heart failure hospitalization, with consistent effects across subgroups. No increase in conduction disorders or other adverse outcomes was observed. These findings suggest potential antiarrhythmic properties of memantine without proarrhythmic signals, warranting mechanistic exploration and confirmation in prospective randomized studies.

### Ethics statement

As the study was conducted retrospectively and patients were not directly involved in its conduct, there was no impact on their care. Ethical approval was not required, as all data were anonymized.
